# Trispyrazol-1-ylmethane

**DOI:** 10.1107/S1600536808041767

**Published:** 2008-12-13

**Authors:** Tobias Kerscher, Philipp Pust, Richard Betz, Peter Klüfers, Peter Mayer

**Affiliations:** a Ludwig-Maximilians Universität, Department Chemie und Biochemie, Butenandtstrasse 5–13 (Haus D), 81377 München, Germany

## Abstract

In the title compound, C_10_H_10_N_6_, the three N atoms in the 2-positions of the pyrazole rings (the ones not bridging to the central C atom are acceptors for weak C—H⋯N contacts with H⋯N distances ranging from 2.49 to 2.59 Å). These furnish the formation of layers perpendicular to [100]. An ortho­rhom­bic polymorph of the title compound has already been described [McLauchlan *et al.* (2004 ▶). *Acta Cryst.* E**60**, o1419–o1420].

## Related literature

The compound was prepared according to a published procedure (Reger *et al.*, 2000 ▶). For a structure analysis of the ortho­rhom­bic polymorph, see: McLauchlan *et al.* (2004 ▶). For classification of hydrogen bonds, see: Bernstein *et al.* (1995 ▶); Etter *et al.* (1990 ▶).
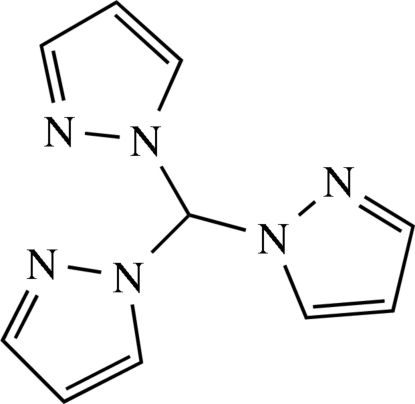


## Experimental

### 

#### Crystal data

C_10_H_10_N_6_
*M* *_r_* = 214.24
Triclinic, 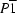

*a* = 7.7216 (9) Å
*b* = 7.8946 (6) Å
*c* = 9.4143 (10) Å
α = 99.292 (8)°
β = 100.023 (9)°
γ = 107.045 (9)°
*V* = 526.36 (10) Å^3^
*Z* = 2
Mo *K*α radiation
μ = 0.09 mm^−1^
*T* = 200 (2) K
0.34 × 0.20 × 0.14 mm

#### Data collection

Nonius KappaCCD diffractometer
Absorption correction: analytical (de Meulenaer & Tompa, 1965 ▶) *T*_min_ = 0.975, *T*_max_ = 0.989
4340 measured reflections
2117 independent reflections
1054 reflections with *I* > 2σ(*I*)
*R*_int_ = 0.030

#### Refinement

*R*[*F* ^2^ > 2σ(*F* ^2^)] = 0.037
*wR*(*F* ^2^) = 0.082
*S* = 0.83
2117 reflections
145 parameters
H-atom parameters constrained
Δρ_max_ = 0.13 e Å^−3^
Δρ_min_ = −0.18 e Å^−3^

### 

Data collection: *CrysAlis CCD* (Oxford Diffraction, 2005 ▶); cell refinement: *CrysAlis RED* (Oxford Diffraction, 2005 ▶); data reduction: *CrysAlis RED*; program(s) used to solve structure: *SHELXS97* (Sheldrick, 2008 ▶); program(s) used to refine structure: *SHELXL97* (Sheldrick, 2008 ▶); molecular graphics: *ORTEP-3* (Farrugia, 1997 ▶) and *Mercury* (Macrae *et al*., 2006 ▶); software used to prepare material for publication: *SHELXL97*.

## Supplementary Material

Crystal structure: contains datablocks global, I. DOI: 10.1107/S1600536808041767/bt2828sup1.cif

Structure factors: contains datablocks I. DOI: 10.1107/S1600536808041767/bt2828Isup2.hkl

Additional supplementary materials:  crystallographic information; 3D view; checkCIF report

## Figures and Tables

**Table 1:** Hydrogen-bond geometry (Å, °)

*D*—H⋯*A*	*D*—H	H⋯*A*	*D*⋯*A*	*D*—H⋯*A*
C10—H10⋯N6^i^	1.00	2.49	3.451 (2)	161
C6—H6⋯N2^ii^	0.95	2.51	3.432 (2)	163
C1—H1⋯N4^iii^	0.95	2.59	3.353 (2)	138

Symmetry codes: (i) 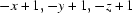; (ii) 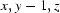; (iii) 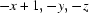.

**Fig. 1.**
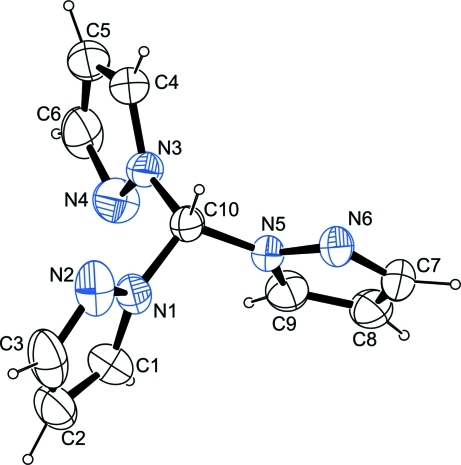
The molecular structure of the title compound with atom labels and anisotropic displacement ellipsoids (drawn at 50% probability level) for non-H atoms.

**Fig. 2.**
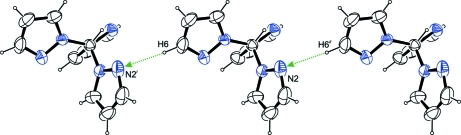
Strands formed by intermolecular C–H···N contacts in the crystal structure of the title compound, viewed along [1 0 0]. Symmetry codes: ix, y - 1, z; iix, y + 1, z.

**Fig. 3.**
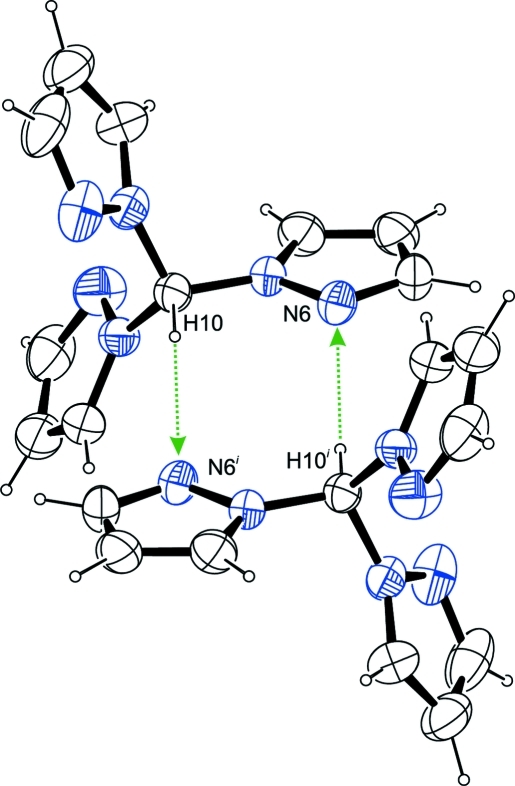
Dimeric units in the crystal structure of the title compound, formed by intermolecular C–H···N contacts whose ranges fall by about 0.2 Å below the sum of van der Waals radii of the corresponding atoms, viewed along [0 1 0]. Symmetry code: i -x + 1, -y + 1, -z + 1.

**Fig. 4.**
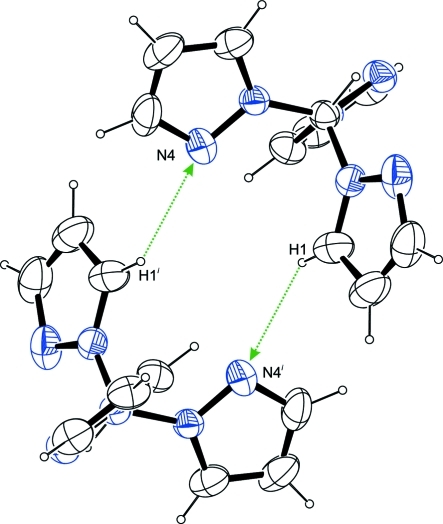
Dimeric units in the crystal structure of the title compound, formed by intermolecular C–H···N contacts whose ranges fall by about 0.1 Å below the sum of van der Waals radii of the corresponding atoms, viewed along [0 1 0]. Symmetry code: i -x + 1, -y, -z.

**Fig. 5.**
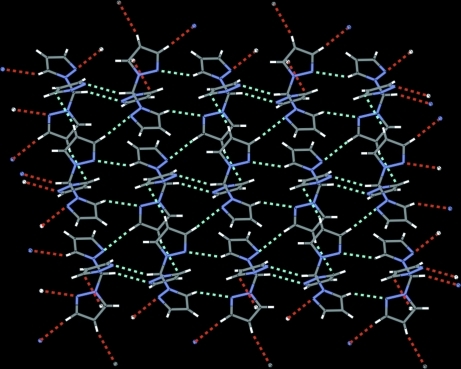
Schematic presentation of the layers formed by C—H···N contacts. Viewing direction approximately along [1 0 0].

**Fig. 6.**
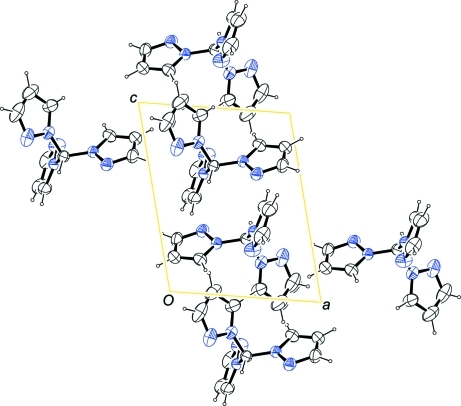
The packing of the title compound, viewed along [0 1 0].

## supplementary crystallographic information

### Comment

The title compound was synthesized as a neutral tridentate ligand for coordination studies with transition metals.

In the molecule, three pyrazole moieties are *N*-bound to a central C atom (Fig. 1). The molecule is found in a non-symmetric conformation in the solid state. The highest possible symmetry *C*_3_*_v_* is broken by the ring containing N4 which is flipped by about 180°.

If only such contacts whose range falls by about 0.2 Å below the sum of van der Waals radii are considered, the crystal structure shows two C–H···N contacts. Infinite strands along [0 1 0] are formed by C6—H6···N2 contacts (Fig. 2). This pattern can be described according to graph-set analysis (Etter *et al.*, 1990; Bernstein *et al.*, 1995) with a C(7) descriptor on the unitary level. In addition, dimeric units are formed by interaction of the H atom of C10 and N6 (Fig. 3). These dimers can be described with a *R*^2^_2_(8) descriptor on the unitary level. Both these described interactions give rise to tubes along [0 1 0].

Considering also contacts whose range falls below the sum of van der Waals radii by only about 0.1 Å, a second dimeric ring system is obvious with a *R*^2^_2_(12) descriptor formed by the H atom of C1 and N4 (Fig. 4). In combination with the interactions described above, this second ring system features the formation of layers perpendicular to [1 0 0] (Fig. 5).

The molecular packing is shown in Figure 6.

### Experimental

The compound was prepared according to a published procedure (Reger *et al.*, 2000) upon reaction of pyrazole and chloroform in alkaline aqueous media in the presence of a phase-transfer catalyst (tetrabutylammonium chloride).

### Refinement

Carbon-bound H atoms were placed in calculated positions (C—H 1.00 Å for the tertiary C atom and C—H 0.95 Å for aromatic C atoms) and were included in the refinement in the riding model approximation, with *U*(H) set to 1.2*U*_eq_(C).

### Crystal data

**Table:**

C_10_H_10_N_6_	*Z* = 2
*M**_r_* = 214.24	*F*(000) = 224
Triclinic, *P*1	*D*_x_ = 1.352 Mg m^−^^3^
Hall symbol: -P 1	Mo *K*α radiation, λ = 0.71073 Å
*a* = 7.7216 (9) Å	Cell parameters from 1494 reflections
*b* = 7.8946 (6) Å	θ = 3.9–26.3°
*c* = 9.4143 (10) Å	µ = 0.09 mm^−^^1^
α = 99.292 (8)°	*T* = 200 K
β = 100.023 (9)°	Block, colourless
γ = 107.045 (9)°	0.34 × 0.20 × 0.14 mm
*V* = 526.36 (10) Å^3^	

### Data collection

**Table:**

Nonius KappaCCD diffractometer	2117 independent reflections
Radiation source: fine-focus sealed tube	1054 reflections with *I* > 2σ(*I*)
graphite	*R*_int_ = 0.030
ω scans	θ_max_ = 26.3°, θ_min_ = 3.9°
Absorption correction: analytical (de Meulenaer & Tompa, 1965)	*h* = −9→9
*T*_min_ = 0.975, *T*_max_ = 0.989	*k* = −9→9
4340 measured reflections	*l* = −11→11

### Refinement

**Table:**

Refinement on *F*^2^	Primary atom site location: structure-invariant direct methods
Least-squares matrix: full	Secondary atom site location: difference Fourier map
*R*[*F*^2^ > 2σ(*F*^2^)] = 0.037	Hydrogen site location: inferred from neighbouring sites
*wR*(*F*^2^) = 0.082	H-atom parameters constrained
*S* = 0.83	*w* = 1/[σ^2^(*F*_o_^2^) + (0.04*P*)^2^] where *P* = (*F*_o_^2^ + 2*F*_c_^2^)/3
2117 reflections	(Δ/σ)_max_ < 0.001
145 parameters	Δρ_max_ = 0.13 e Å^−^^3^
0 restraints	Δρ_min_ = −0.18 e Å^−^^3^

### Fractional atomic coordinates and isotropic or equivalent isotropic displacement parameters (Å^2^)

**Table:**

	*x*	*y*	*z*	*U*_iso_*/*U*_eq_	
N5	0.36455 (16)	0.27051 (16)	0.27731 (13)	0.0334 (3)	
N1	0.64066 (18)	0.38873 (17)	0.19270 (15)	0.0379 (3)	
N6	0.28341 (18)	0.38479 (16)	0.33973 (14)	0.0409 (4)	
N3	0.63243 (17)	0.18619 (16)	0.35571 (14)	0.0367 (3)	
C10	0.5661 (2)	0.32745 (19)	0.31338 (16)	0.0334 (4)	
H10	0.6125	0.4332	0.4005	0.040*	
N2	0.78449 (19)	0.54772 (17)	0.22761 (17)	0.0539 (4)	
N4	0.6002 (2)	0.02927 (18)	0.25599 (16)	0.0517 (4)	
C7	0.1030 (2)	0.2989 (2)	0.28350 (19)	0.0491 (5)	
H7	0.0086	0.3453	0.3072	0.059*	
C4	0.7280 (2)	0.1888 (2)	0.49067 (19)	0.0489 (5)	
H4	0.7652	0.2845	0.5766	0.059*	
C9	0.2372 (2)	0.1197 (2)	0.18445 (18)	0.0444 (4)	
H9	0.2619	0.0219	0.1283	0.053*	
C8	0.0671 (2)	0.1351 (2)	0.18683 (18)	0.0497 (5)	
H8	−0.0510	0.0511	0.1335	0.060*	
C6	0.6797 (3)	−0.0647 (2)	0.3351 (2)	0.0582 (5)	
H6	0.6807	−0.1830	0.2961	0.070*	
C1	0.5986 (3)	0.3080 (2)	0.0478 (2)	0.0535 (5)	
H1	0.5039	0.1956	−0.0004	0.064*	
C2	0.7163 (3)	0.4167 (3)	−0.0163 (2)	0.0641 (6)	
H2	0.7215	0.3975	−0.1175	0.077*	
C3	0.8267 (3)	0.5611 (3)	0.0972 (3)	0.0656 (6)	
H3	0.9231	0.6602	0.0842	0.079*	
C5	0.7606 (3)	0.0283 (3)	0.4800 (2)	0.0599 (5)	
H5	0.8254	−0.0117	0.5560	0.072*	

### Atomic displacement parameters (Å^2^)

**Table:**

	*U*^11^	*U*^22^	*U*^33^	*U*^12^	*U*^13^	*U*^23^
N5	0.0307 (8)	0.0385 (8)	0.0336 (8)	0.0143 (6)	0.0090 (7)	0.0083 (6)
N1	0.0393 (8)	0.0377 (8)	0.0436 (9)	0.0164 (6)	0.0167 (7)	0.0141 (7)
N6	0.0413 (9)	0.0479 (8)	0.0409 (9)	0.0237 (7)	0.0141 (7)	0.0087 (7)
N3	0.0416 (8)	0.0411 (8)	0.0325 (8)	0.0213 (6)	0.0091 (7)	0.0085 (7)
C10	0.0334 (9)	0.0360 (9)	0.0325 (9)	0.0149 (7)	0.0079 (8)	0.0062 (7)
N2	0.0499 (9)	0.0383 (9)	0.0822 (12)	0.0167 (7)	0.0294 (9)	0.0183 (8)
N4	0.0703 (10)	0.0433 (9)	0.0505 (10)	0.0312 (8)	0.0175 (8)	0.0085 (8)
C7	0.0349 (11)	0.0708 (13)	0.0519 (12)	0.0241 (9)	0.0156 (9)	0.0242 (10)
C4	0.0463 (11)	0.0698 (13)	0.0410 (11)	0.0276 (9)	0.0133 (9)	0.0221 (9)
C9	0.0423 (11)	0.0436 (11)	0.0377 (11)	0.0079 (9)	0.0037 (9)	0.0004 (8)
C8	0.0372 (11)	0.0635 (13)	0.0390 (11)	0.0066 (9)	0.0030 (9)	0.0105 (9)
C6	0.0707 (13)	0.0529 (12)	0.0802 (16)	0.0404 (11)	0.0395 (13)	0.0348 (12)
C1	0.0595 (12)	0.0647 (12)	0.0408 (11)	0.0214 (10)	0.0187 (10)	0.0154 (10)
C2	0.0760 (15)	0.0869 (15)	0.0625 (14)	0.0479 (12)	0.0409 (13)	0.0427 (13)
C3	0.0698 (14)	0.0579 (13)	0.0996 (18)	0.0327 (11)	0.0552 (15)	0.0422 (13)
C5	0.0639 (13)	0.0855 (15)	0.0598 (14)	0.0454 (11)	0.0279 (12)	0.0449 (12)

### Geometric parameters (Å, °)

**Table:**

N5—C9	1.3516 (18)	C7—H7	0.9500
N5—N6	1.3565 (15)	C4—C5	1.354 (2)
N5—C10	1.4475 (18)	C4—H4	0.9500
N1—C1	1.348 (2)	C9—C8	1.357 (2)
N1—N2	1.3562 (16)	C9—H9	0.9500
N1—C10	1.4486 (17)	C8—H8	0.9500
N6—C7	1.3243 (19)	C6—C5	1.378 (2)
N3—C4	1.3473 (19)	C6—H6	0.9500
N3—N4	1.3566 (16)	C1—C2	1.351 (2)
N3—C10	1.4397 (18)	C1—H1	0.9500
C10—H10	1.0000	C2—C3	1.373 (3)
N2—C3	1.336 (2)	C2—H2	0.9500
N4—C6	1.330 (2)	C3—H3	0.9500
C7—C8	1.378 (2)	C5—H5	0.9500
			
C9—N5—N6	111.92 (13)	C5—C4—H4	126.7
C9—N5—C10	130.33 (14)	N5—C9—C8	106.78 (15)
N6—N5—C10	117.71 (12)	N5—C9—H9	126.6
C1—N1—N2	112.14 (13)	C8—C9—H9	126.6
C1—N1—C10	130.61 (13)	C9—C8—C7	105.04 (15)
N2—N1—C10	117.11 (13)	C9—C8—H8	127.5
C7—N6—N5	103.53 (12)	C7—C8—H8	127.5
C4—N3—N4	112.51 (13)	N4—C6—C5	112.70 (17)
C4—N3—C10	126.82 (14)	N4—C6—H6	123.7
N4—N3—C10	120.67 (13)	C5—C6—H6	123.7
N3—C10—N5	111.58 (11)	N1—C1—C2	107.29 (16)
N3—C10—N1	111.25 (11)	N1—C1—H1	126.4
N5—C10—N1	111.28 (12)	C2—C1—H1	126.4
N3—C10—H10	107.5	C1—C2—C3	104.79 (17)
N5—C10—H10	107.5	C1—C2—H2	127.6
N1—C10—H10	107.5	C3—C2—H2	127.6
C3—N2—N1	102.69 (15)	N2—C3—C2	113.08 (16)
C6—N4—N3	102.95 (14)	N2—C3—H3	123.5
N6—C7—C8	112.73 (15)	C2—C3—H3	123.5
N6—C7—H7	123.6	C4—C5—C6	105.23 (17)
C8—C7—H7	123.6	C4—C5—H5	127.4
N3—C4—C5	106.61 (16)	C6—C5—H5	127.4
N3—C4—H4	126.7		
			
C9—N5—N6—C7	0.54 (15)	C10—N3—N4—C6	−179.60 (13)
C10—N5—N6—C7	178.36 (12)	N5—N6—C7—C8	−0.47 (17)
C4—N3—C10—N5	−113.15 (16)	N4—N3—C4—C5	0.17 (17)
N4—N3—C10—N5	66.40 (16)	C10—N3—C4—C5	179.76 (13)
C4—N3—C10—N1	121.92 (15)	N6—N5—C9—C8	−0.41 (17)
N4—N3—C10—N1	−58.53 (17)	C10—N5—C9—C8	−177.88 (13)
C9—N5—C10—N3	−51.4 (2)	N5—C9—C8—C7	0.10 (17)
N6—N5—C10—N3	131.27 (13)	N6—C7—C8—C9	0.24 (19)
C9—N5—C10—N1	73.53 (18)	N3—N4—C6—C5	−0.21 (18)
N6—N5—C10—N1	−103.82 (13)	N2—N1—C1—C2	−0.57 (19)
C1—N1—C10—N3	73.6 (2)	C10—N1—C1—C2	−176.15 (15)
N2—N1—C10—N3	−101.77 (14)	N1—C1—C2—C3	0.2 (2)
C1—N1—C10—N5	−51.5 (2)	N1—N2—C3—C2	−0.5 (2)
N2—N1—C10—N5	133.13 (13)	C1—C2—C3—N2	0.2 (2)
C1—N1—N2—C3	0.65 (17)	N3—C4—C5—C6	−0.28 (18)
C10—N1—N2—C3	176.88 (13)	N4—C6—C5—C4	0.3 (2)
C4—N3—N4—C6	0.02 (16)		

### Hydrogen-bond geometry (Å, °)

**Table:**

*D*—H···*A*	*D*—H	H···*A*	*D*···*A*	*D*—H···*A*
C10—H10···N6^i^	1.00	2.49	3.451 (2)	161
C6—H6···N2^ii^	0.95	2.51	3.432 (2)	163
C1—H1···N4^iii^	0.95	2.59	3.353 (2)	138

Symmetry codes: (i) −*x*+1, −*y*+1, −*z*+1; (ii) *x*, *y*−1, *z*; (iii) −*x*+1, −*y*, −*z*.
